# Ligandbook: an online repository for small and drug-like molecule force field parameters

**DOI:** 10.1093/bioinformatics/btx037

**Published:** 2017-01-27

**Authors:** Jan Domański, Oliver Beckstein, Bogdan I Iorga

**Affiliations:** 1 Department of Biochemistry, University of Oxford, Oxford, UK; 2 Laboratory of Chemical Physics, National Institute of Diabetes and Digestive and Kidney Diseases, National Institutes of Health, Bethesda, MD, USA; 3 Department of Physics, Arizona State University, Tempe, AZ, USA; 4 Center for Biological Physics, Arizona State University, Tempe, AZ, USA; 5 Institut de Chimie des Substances Naturelles, CNRS UPR 2301, Université Paris-Saclay, Labex LERMIT, Gif-sur-Yvette, France

## Abstract

**Summary:**

Ligandbook is a public database and archive for force field parameters of small and drug-like molecules. It is a repository for parameter sets that are part of published work but are not easily available to the community otherwise. Parameter sets can be downloaded and immediately used in molecular dynamics simulations. The sets of parameters are versioned with full histories and carry unique identifiers to facilitate reproducible research. Text-based search on rich metadata and chemical substructure search allow precise identification of desired compounds or functional groups. Ligandbook enables the rapid set up of reproducible molecular dynamics simulations of ligands and protein-ligand complexes.

**Availability and Implementation:**

Ligandbook is available online at https://ligandbook.org and supports all modern browsers. Parameters can be searched and downloaded without registration, including access through a programmatic RESTful API. Deposition of files requires free user registration. Ligandbook is implemented in the PHP Symfony2 framework with TCL scripts using the CACTVS toolkit.

**Supplementary information:**

Supplementary data are available at *Bioinformatics* online.

## 1 Introduction

The computational drug design process relies on the parameterization of atomic interactions through empirical force fields in order to probe protein–drug interactions with classical molecular dynamics or Monte Carlo simulations. The chemical diversity of drug-like molecules makes it difficult to generate reliable and transferable parameter sets for these compounds, compared to established parameters for, e.g. proteins or water. High-quality parameterizations of small molecules require specialist knowledge and the shortage of such expertise—and hence lack of high-quality parameters—presents a barrier for the field.

Servers and tools exist to parameterize small molecules on demand such as *ParamChem* (Vanommeslaeghe and MacKerell, 2012), *SwissParam* (Zoete *et al.*, 2011), the *Antechamber* tool (Case *et al.*, 2005), the *R.E.DD.B.* server (Dupradeau *et al.*, 2008) and *Automated Topology Builder* (ATB) and Repository (Malde *et al.*, 2011). However, a reference repository for small molecule parameters generated by other means and especially for ones published in the literature is lacking. Such published parameters are valuable because they have typically been validated and can be easily used in new studies but they can be difficult to find and are often invisible to most searches, e.g. as part of supplementary information. Only a few, very specialized repositories for force field parameters exist: The *Gromacs molecule & liquid database* (Fischer *et al.*, 2015; van der Spoel *et al.*, 2012) contains about 500 parameter sets (OPLS-AA, AMBER/GAFF and CHARMM/CGenFF for Gromacs (Abraham *et al.*, 2015)) for more than 150 organic molecules together with experimental liquid properties for validation purposes (as well as thermodynamic gas phase properties for about 3000 molecules (Ghahremanpour *et al.*, 2016)). Our own *Lipidbook* repository (Domański *et al.*, 2010) archives lipid and detergent force field parameters since 2009 and is a successful example for how a community-curated repository can be reliably maintained over many years.

Our new *Ligandbook* site is a public database for force-field parameters of small and drug-like molecules for all major all-atom force fields, including the popular OPLS-AA, CHARMM/CGenFF and AMBER/GAFF varieties. Ligandbook aims to enable parameter re-use and simulation reproducibility by (i) facilitating the publication of force field parameters as open data; (ii) acting as an archive for parameter sets that are supplied and maintained by the community; (iii) making large, richly annotated parameter datasets easily available through human and machine accessible interfaces.

## 2 Repository architecture

A set of parameters contained in the repository is called a *package*. For each tautomeric and ionization state of a molecule many packages can be created. A package contains a coordinate file and a topology file with the force field parameters. All files are versioned and each package-version pair has a unique and persistent package identification number. Metadata annotations as well as an abstract are stored to enable rich searching and filtering of packages. A package is linked to a user-supplied citation and carries a license. Ligandbook uses the Cactvs toolkit (Ihlenfeldt *et al.*, 1994) for the underlying cheminformatics functionality (see Implementation in Supplementary Information for details).

## 3 Capabilities

*Search and Download*. The repository can be searched using either text-based or structure-based queries. Text queries can contain words, phrases, wildcards and groupings using boolean operators as well as the advanced Apache Lucene syntax. By default, the text search is performed across all annotation types (including compound names, synonyms and abstract words) but can be limited to a single attribute (for instance, a PDB ligand id or the packageId). For chemical exact structure or substructure search, the query can be drawn interactively in the Cactvs Sketcher (Ihlenfeldt *et al.*, 2009) or entered as a SMILES string.

Each package is shown with all files in its history (with SHA1 checksums), chemical structure depictions and the meta data with links to other databases. Older versions are always available and provide a transparent history of changes so that studies can be reproduced with the exact same parameters. A package can be downloaded as a zip file containing the files together with the license governing use of the parameters. References associated with the parameterizations are included so that users can ascertain appropriate use and cite the original authors appropriately. Additionally, if provided by the depositor, computed values and reference values for validation observables are shown as well as a subjective reliability score from 1 (not validated or not reliable) to 5 (very reliable); see Supplementary Information, Data structures and versioning.

The database can also be queried directly with 3D coordinates. The input structure will be returned with the atoms reordered to match any found parameter files for immediate use in a simulation.

*Programmatic access*. Results can be retrieved in YAML, XML and JSON formats for further automatic downstream processing through a RESTful API with URL-based queries.

*Package deposition*. Because packages are curated and owned by individual users, package creation requires free registration with a valid email address. Upon submission, the uploaded coordinate and topology files are processed, checked if they can be parsed by the Gromacs grompp input processor (Abraham *et al.*, 2015), and a preview of the data is shown to the user for approval or corrections. Most of the meta data are automatically derived from the chemical structure. Users may supply a description in the abstract field and link the parameters to publications, which can be automatically fetched via PubMed IDs, as well as submit computed and reference validation values and provide a subjective reliability rating together with a free-form justification. Users must accept the CC BY-SA (Creative Commons Attribution-ShareAlike) license for the parameters and the CC0 Public Domain Dedication for all meta data (see Supplementary Information, Licenses). The package author may update some or all files, which creates a new version of the package.

## 4 Initial content

Currently the repository contains more than 2900 packages, formatted for use with Gromacs (Abraham *et al.*, 2015). These include 455 parameter sets that were validated with hydration free energy calculations as part of the SAMPL challenges (Beckstein and Iorga, 2012; Beckstein *et al.*, 2014; Kenney *et al.*, 2016), parameters from some of our previous studies (Simmons *et al.*, 2014), and > 2000 packages with ligands from the PDB, parameterized with mol2ff (manuscript in preparation). Because many common drugs are already included, users can easily set up simulations that probe protein-drug interactions (see Supplementary Information for an example).

Coordinates are provided as pdb files. Topologies are always present as Gromacs itp files (Abraham *et al.*, 2015) although files in other formats can be optionally deposited as supplementary data, such as CHARMM prm files for CHARMM/CGenFF parameter sets. If necessary, itp files can be converted into or from other commonly used file formats (AMBER, CHARMM, Desmond, LAMMPS, etc.) using open source tools such as InterMol (https://github.com/shirtsgroup/InterMol), ParmEd (https://github.com/ParmEd/ParmEd), or acpype (Sousa da Silva and Vranken, 2012).

## 5 Conclusions

Ligandbook provides the infrastructure for a reference repository for ligand topologies and parameters. It is designed for growth and for wide use by the community. With its focus on open data and interoperability, it provides opportunities for other researchers to tap into a large and growing dataset of parameterizations. Ligandbook should become useful in the development of automated parameterization methods and for molecular simulations of drug-protein interactions.

## Supplementary Material

Supplementary Data

## References

[btx037-B1] Abraham M.J. et al (2015) GROMACS: high performance molecular simulations through multi-level parallelism from laptops to supercomputers. SoftwareX, 1–2, 19–25.

[btx037-B2] Beckstein O., Iorga B.I. (2012) Prediction of hydration free energies for aliphatic and aromatic chloro derivatives using molecular dynamics simulations with the OPLS-AA force field. J. Comput. Aided Mol. Des., 26, 635–645. 22187140 10.1007/s10822-011-9527-9

[btx037-B3] Beckstein O. et al (2014) Prediction of hydration free energies for the SAMPL4 diverse set of compounds using molecular dynamics simulations with the OPLS-AA force field. J. Comput. Aided Mol. Des., 28, 265–276. 24557853 10.1007/s10822-014-9727-1

[btx037-B4] Case D. et al (2005) The Amber biomolecular simulation programs. J. Comput. Chem., 26, 1668–1688. 16200636 10.1002/jcc.20290 PMC1989667

[btx037-B5] Domański J. et al (2010) Lipidbook: a public repository for force-field parameters used in membrane simulations. J. Membr. Biol., 236, 255–258. 20700585 10.1007/s00232-010-9296-8

[btx037-B6] Dupradeau F. et al (2008) R.E.DD.B.: A database for RESP and ESP atomic charges, and force field libraries. Nucleic Acids Res., 36, D360–D367. 17962302 10.1093/nar/gkm887 PMC2238896

[btx037-B7] Fischer N.M. et al (2015) Properties of organic liquids when simulated with long-range Lennard-Jones interactions. J. Chem. Theory Comput., 11, 2938–2944. 26575731 10.1021/acs.jctc.5b00190

[btx037-B8] Ghahremanpour M.M. et al (2016) Large-scale calculations of gas phase thermochemistry: Enthalpy of formation, standard entropy, and heat capacity. J. Chem. Phys., 145, 114305.

[btx037-B9] Ihlenfeldt W. et al (1994) Computation and management of chemical properties in CACTVS: An extensible networked approach toward modularity and compatibility. J. Chem. Inf. Comput. Sci., 34, 109–116.

[btx037-B10] Ihlenfeldt W. et al (2009) The PubChem chemical structure sketcher. J. Cheminform., 1, 20. 20298522 10.1186/1758-2946-1-20 PMC2820498

[btx037-B11] Kenney I.M. et al (2016) Prediction of cyclohexane-water distribution coefficients for the SAMPL5 data set using molecular dynamics simulations with the OPLS-AA force field. J. Comput. Aided Mol. Des., 30, 1045–1058. 27581968 10.1007/s10822-016-9949-5

[btx037-B12] Malde A. et al (2011) An automated force field topology builder (ATB) and repository: Version 1.0. J. Chem. Theory Comput., 7, 4026–4037. 26598349 10.1021/ct200196m

[btx037-B13] Simmons K.J. et al (2014) The molecular mechanism of ligand recognition by membrane transport protein, Mhp1. EMBO J., 33, 1831–1844. 24952894 10.15252/embj.201387557 PMC4195764

[btx037-B14] Sousa da Silva A.W., Vranken W.F. (2012) ACPYPE – AnteChamber PYthon Parser interfacE. BMC Res. Notes, 5, 367. 22824207 10.1186/1756-0500-5-367 PMC3461484

[btx037-B15] van der Spoel D. et al (2012) GROMACS molecule & liquid database. Bioinformatics, 28, 752–753. 22238269 10.1093/bioinformatics/bts020

[btx037-B16] Vanommeslaeghe K., MacKerell A.D. (2012) Automation of the CHARMM General Force Field (CGenFF) I: bond perception and atom typing. J. Chem. Inf. Model., 52, 3144–3154. 23146088 10.1021/ci300363c PMC3528824

[btx037-B17] Zoete V. et al (2011) SwissParam: a fast force field generation tool for small organic molecules. J. Comput. Chem., 32, 2359–2368. 21541964 10.1002/jcc.21816

