# Supplementary material for: Ligandbook: an online repository for small and drug-like molecule force field parameters
Source: Bioinformatics. 2017 Jan 27;33(11):1747–9. doi: 10.1093/bioinformatics/btx037 (PMC5447236; doi:10.1093/bioinformatics/btx037)
Supplement: Supplementary Data [file btx037_supp.pdf]

Supplementary Information
Pages 1–9

Supplementary Information for: Ligandbook — an online repository for small and drug-like molecule force field parameters

Jan Domański [1,2], Oliver Beckstein [3,4,*] and Bogdan I. Iorga [5,*]

[1]Department of Biochemistry, University of Oxford, South Parks Road, Oxford, OX1 3QU, United Kingdom
[2]Laboratory of Chemical Physics, National Institute of Diabetes and Digestive and Kidney Diseases, National Institutes of Health, Bethesda, Maryland 20892-0520, United States
[3]Department of Physics, Arizona State University, Tempe, AZ 85287-1504, United States
[4]Center for Biological Physics, Arizona State University, Tempe, AZ 85287-1504, United States
[5]Institut de Chimie des Substances Naturelles, CNRS UPR 2301, Université Paris-Saclay, Labex LERMIT, 91198 Gif-sur-Yvette, France

The Supplementary Information provides additional details on the implementation and web site structure of *Ligandbook*. It also introduces the main data structures and describes the programmatic API. The search functionality is explained with examples and a tutorial shows how easy it is to simulate drug-protein interactions using data provided by Ligandbook.

1 IMPLEMENTATION DETAILS

Ligandbook is written in PHP with the Symfony2 framework. It stores object annotations in a MYSQL relational database while deposited files use filesystem-backed storage (see Supplementary Information Fig. 1). The PHP code interfaces with TCL scripts that use the CACTVS toolkit (Ihlenfeldt *et al.*, 1994) for the cheminformatics functionality. Chemical structures for structure-based queries are drawn with the CACTVS Sketcher (Ihlenfeldt *et al.*, 2009). The text search uses the Elasticsearch engine (`https://github.com/elastic/elasticsearch`). Elastica indexes are created for a range of information, including but not exclusive to: canonical IUPAC name, common names, formula, molecular weight, charge, number of atoms, SMILES, PubChem CID, PDB ligand ID, CAS RN. Indexing these fields permits rapid sifting and searching of the repository.

The site `https://ligandbook.org` runs on a Linux server with eight cores (dual Intel Xeon CPU E5506, 2.13GHz) under the CentOS 7.2 operating system. It uses the APACHE 2.4 webserver (`https://httpd.apache.org/`) and MYSQL 5.6 (`https://www.mysql.com/`) as a relational database.

The security of the server and of data contained is ensured by secured connections (SSL encryption), use of CAPTCHA for user identification and email address validation when the user account is created. User passwords are salted and hashed with a functionality provided by Symfony2. The access to database objects is restricted via the use of Access Control Lists (ACLs) that define the user "OWNER" of a particular resource. Other users commonly have only "VIEW" permissions. Ligandbook maintainers have "ADMIN" permissions, with access to all data structures.

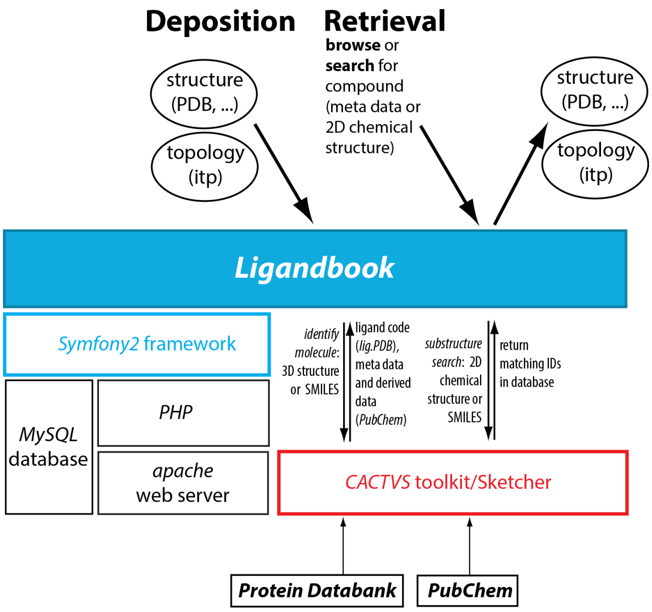

Fig. 1: Software architecture and information flow. Users interact through the *Ligandbook* web frontend to deposit parameter files or query the database to retrieve parameter files (structures and topologies). Internally, *Ligandbook* uses the CACTVS toolkit to process chemical structures.

*to whom correspondence should be addressed: oliver.beckstein@asu.edu (OB), bogdan.iorga@cnrs.fr (BII)

© The Authors 2016.

1.1 Software architecture

The software architecture of the Ligandbook repository is represented in Figure 1. The user-facing web frontend is built with the Symfony2 PHP framework (`https://symfony.com/`). Symfony2 provides high-quality, maintainable, and upgradable components for web applications following the model-view-controller (MVC) pattern. By using a reliable foundation, we are able to maintain the site for the long term at low costs and with low developer overhead. For instance, security updates can be easily incorporated. Symfony also comes with numerous components such as authentication, emailer (for registration and password recovery emails), a rich text editor widget, and an admin backend that are all used inside Ligandbook. Symfony2 also abstracts the access to the underlying MYSQL database. The CACTVS cheminformatics toolkit (Ihlenfeldt *et al.*, 1994) (`http://www.xemistry.com/`) provides the functionality to identify compounds from 3D structures, perform substructure search, generate 2D structure images, and fetch metadata from PubChem. The PHP code in the web frontend communicates to CACTVS through scripts written in TCL.

The text search is performed with the Elasticsearch engine, which is built with the Apache Lucene Core search engine library (McCandless *et al.*, 2010). Therefore, our text search supports the advanced Apache Lucene syntax with Boolean operators, grouping, wildcards, fuzzy search, and search by specific fields.

Users interact with the underlying database through the web frontend. A guiding principle of Ligandbook is that chemical structures are accurately represented and the whole site is "chemically aware", i.e. compounds can be searched by structure and are identified based on their chemistry. On deposition, the 3D coordinate file submitted by the user is parsed and represented as a 2D chemical structure. This step enables the user to spot and correct any mistakes that can occur when deriving bond order information from 3D structures. The chemical structure is then used to derive most of the meta data and to link to PubChem and to other data bases. The most accurate way to query the database is the chemical search whereby the user draws the 2D chemical structure (or provides a SMILES string to have the structure drawn).

The chemical structure is also required to automatically generate the chemical structure depictions (in SVG format). Two depictions are made available as zoomable images. A simplified representations only shows the heteroatoms explicitly. A detailed representation includes all atoms and labels each atom explicitly, which aids in identifying atoms within structure and topology files.

1.2 Data structures and versioning

Compounds Ligandbook associates an individual molecule, here named a *compound*, with meta data about the molecule itself and user-provided data. Compounds are described by their chemical 2D structure. Compound details are generated by CACTVS or fetched from external sources. Each compound is uniquely identified with a hash key (HASHISY) as generated by CACTVS. If available, the compound name is taken, in order of availability, from the first CommonName, the IUPAC name or the PDB.

In order to facilitate validation of of parameter sets, an arbitrary number of *reference values* can be associated with a compound. A reference value is an observable such as an experimental hydration free energy or a high-level quantum mechanical calculation of a torsional energy barrier. Each reference value consists of a text description, a numerical value, the units, and a literature citation.

Table 1. Reliability rating. The rating is optional and chosen by the depositor of a parameter set.

rating	meaning
1	completely unvalidated, the parameters run in a simulation, but no properties of the system have been validated
2	minimally validated, structural properties are acceptable but no explicit comparison to experiment was done
3	reasonable parameters that agree with 1-2 experimental measurements, major problems are apparent
4	strong agreement between a number of reference and computed properties, minor problems
5	perfect agreement between multiple experimental and computed values

Packages and versions A *package* contains meta data and one or more *versions*. A version is a container for structure and topology files. If there are reference values asscociated with the compound then there should also be corresponding *computed values* stored with a version. These computed values are the values of the reference observable as produced by the particular parameter set contained in the version. Users may compare the computed values to the reference values in order to judge the quality of the parameters. Additionally, the depositor may assign a subjective reliability rating to a version (typically in conjunction with reference and computed values). For simplicity, a simple integer scale from 1 to 5 was adopted, together with a free form text field suitable for a short justification of the rating; the intended meaning of the scale is listed in Table 1.

There can be multiple versions in a package, each with its own unique identifier. The versions make up the history of a parameter set. The files in versions are stored together with SHA1 checksums so that users can immediately identify files that changed between versions. Individual versions of parameters can be accessed through the web interface or through programmatic access (see Section 1.4).

1.3 Site structure

At the top of the Ligandbook home page (Fig. 2a) and all other top level pages contain a menu with the major functionalities:

Home
link to the home page (Fig. 2)

Search
text search or search by 2D structure with the CACTVS Sketcher (see Section 3)

Reorder
search with a 3D structure and return a structure whose atom are reordered so that it can be used with matching topology files found in the repository (see Section 4)

Deposit
submit a new package

Documentation
top level page for documentation

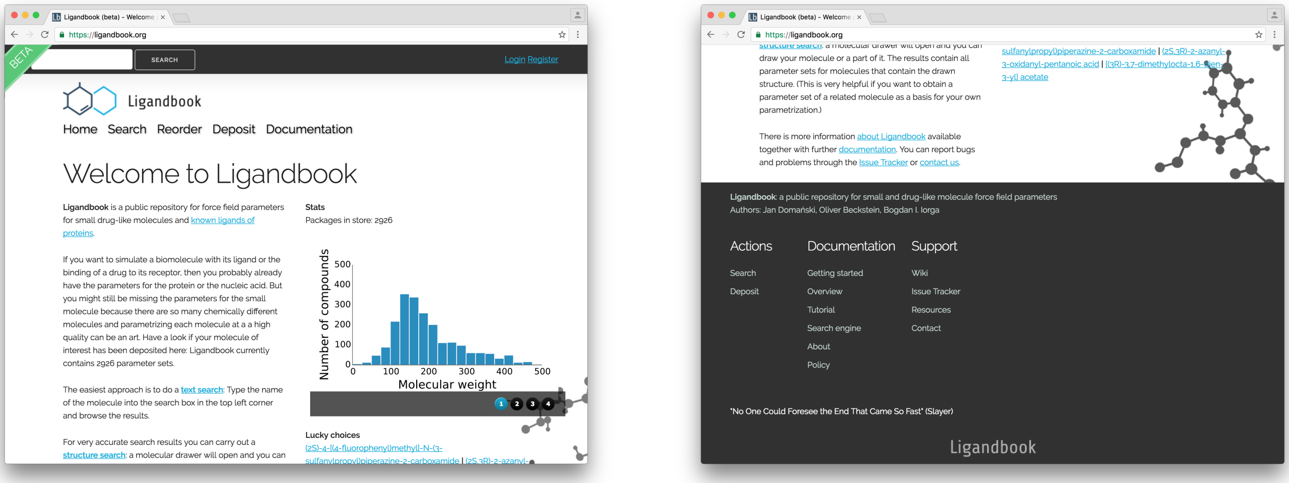

(a) Top of the home page.

(b) Footer of the home page.

Fig. 2: Home page of *Ligandbook*. The site is accessible with all modern browsers using HTTP through SSL. From top (a) to bottom (b): The top bar contains a text search box and links for registration and log in and underneath the main menu. The graphic slider shows various statistics about the molecules in the repository and is updated nightly. The "lucky choices" are randomly generated links to packages in the repository and invite the casual user to browse and explore the site. The footer menus provide links to specific documentation pages, the wiki, and the issue tracker. All pages display the same top bar and main menu as well as the footer menu and thus make it easy for the user to navigate the site.

By default, users can access the site anonymously. In order to deposit a package or in order to curate an existing package, users have to register with a valid email address and log in with their user name and password. The *Register* and *Login* menu items in the top right corner provide this functionality. If users forget their password, they can reset it by themselves and obtain a temporary password through their registered email address.

Further functionality and technical details of the repository are described in more detail in *Documentation* and *Support* sections of the web page that can be directly accessed from the footer of every page (Fig. 2b). The *Getting started* page contains step-by-step tutorials for commonly used operations. A dedicated *Wiki* and an *Issue tracker* are present to facilitate discussion with the community. The *Policy* governing the repository is also clearly spelt out.

1.4 Programmatic access

In order to increase interoperability with other tools and scripts, Ligandbook defines a RESTful API for searching the repository using URL-based queries. Results can be retrieved in YAML, XML and JSON formats for further automatic downstream processing. For instance, a text search for "benzene" is accessed with the URL

```
https://ligandbook.org/search/benzene/results
```

and shows a package listing in the browser. Modifying the URL string by affixing an extension such as *.json* returns a JSON file instead:

```
https://ligandbook.org/search/benzene/results.json
```

or with *.yml* a YAML file is returned:

```
https://ligandbook.org/search/benzene/results.yml
```

which looks similar to the following


```
query: benzene
limit: 1000
page: '1'
packages:
    -
        id: 928
        created_at: '2016-08-21T18:13:02+0200'
        modified_at: '2016-08-21T18:13:02+0200'
        license:
            id: 928
            name: 'Bogdan I. Iorga'
            email: bogdan.iorga@cnrs.fr
            source: 'MOL2FF (http://mol2ff.icsn.cnrs-gif
                 .fr)'
            licensetype:
                id: 2
                name: 'CC BY-SA - Creative Commons
                     Attribution-Share Alike'
                description: 'The Creative Commons
                     Attribution-Share Alike (CC BY-SA)
                     License allows anyone to use,
                     modify, and distribute your work,
                     provided they attribute it to you (
                     e.g. by citing you) and if they
                     distribute it they must do so under
                      the same or a compatible license.
                     '
                link: '/policy/#cc-by-sa'
        metadata:
```

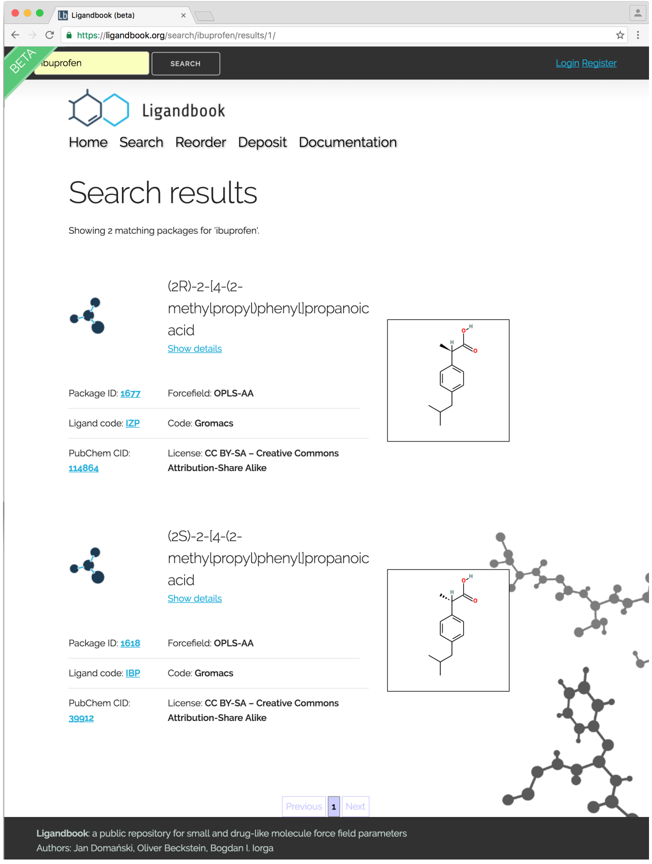

Fig. 3: Text search for *ibuprofen*. The results are shown as a list of package summaries.


```
id: 928
abstract: 'The topology was generated using MOL2FF.'
ligand_code: BNZ
molecule_identifier: 3DB0124A3ECF5ECE
name: BENZENE
canonical_i_u_p_a_cname: benzene
formula: C6H6
molecular_weight: '78.11'
charge: 0
number_of_atoms: 12
_s_m_i_l_e_s: C1=CC=CC=C1
pubchem: 'http://pubchem.ncbi.nlm.nih.gov/summary/summary.cgi?cid=241'
picture:
    id: 3709
    path: svg/57b9d30e3d697.svg
    name: 928-compact.svg
    hash: fef6252ea8e29a4f94bd7dd70be4aeeceb050385

picture_detailed:
    id: 3710
    path: svg/57b9d30e3f0b7.svg
    name: 928-detail.svg
    hash: 9f9f508c1d5582261c9b29d793ddf84b778721a6
    other_names:
        - BENZENE
        - 27271-55-2

        - 'Benzeen [Dutch]'
    _c_a_s_r_n: 71-43-2
versions:
    -
        id: 928
        created_at: '2016-08-21T18:13:02+0200'
        modified_at: '2016-08-21T18:13:02+0200'
        version_id: 1
        version_hash: 593e1df4b55711250e15f4f1c870428d
        topology:
            -
                id: 3711
                path: 57b9d30e3a1e0.itp
                name: BNZ.itp
                hash: 28be89c1d156a75e38915f29d8553b844c0c4d7b

        structure:
            -
                id: 3712
                path: 57b9d30e3bd44.pdb
                name: BNZ.pdb
                hash: 2877b35e97024f98b5ce9e95df682651aa63104e

        download_counter: 0
    code:
        id: 1
        name: Gromacs
        url: nonvalidurl3
        description: 'Gromacs simulation package'
    forcefield:
        id: 1
        name: OPLS-AA
        url: nonvalidurl1
        description: 'OPLS/AA forcefield discription'
    is_valid: true
    is_approved: true
    package_id: 928
-
    id: 935
    created_at: '2016-08-21T18:13:05+0200'
    modified_at: '2016-08-21T18:13:05+0200'
    ...
```


In Python, a query can be easily parsed into a dictionary, using the `yaml` package:

```
import urllib2
import yaml
url = "https://ligandbook.org/search/benzene/results.yml"
response = urllib2.urlopen(url)
results = yaml.load(response)
```

The variable `results` will hold a data structure that contains the complete query. For instance, the total number of packages found is

```
>>> len(results["packages"])
181
```

and the *packageId* of the first package in the list is

```
>>> results["packages"][0]["package_id"]
928
```

Instead of a text search, chemical substructure search can also be accessed through the API. For example, a substructure search for the SMILES `OC(=O)C(C)C1=CC=C(CC(C)C)C=C1` ((2S)-2-[4-(2-methylpropyl)phenyl]propanoic acid, also known as ibuprofen) can be returned in JSON format by using the GET URL

```
https://ligandbook.org/sketch/OC(=O)C(C)C1=CC=C(CC(C)C)C
    =C1/exact_matching/0/results/1/page.json
```

Any GET URL that is produced for a search in Ligandbook can thus be used to access the returned matches in a programmatic fashion.

From the data one can then construct the URL to download the package. Using the benzene example from above, one can use the following Python code to select the first package and then download the contents of the latest version inside the package as a zip file (here just named "download.zip"):

```
package = results["packages"][0]
latest_version = package['versions'][-1]
url = "https://ligandbook.org/package/{0}/version/{1}/
     download".format(package['id'], latest_version['
     id'])
response_zip = urllib2.urlopen(url)
with open("download.zip", "w") as zip_file:
    zip_file.write(response_zip.read())
```

Programmatic access should enable other tools to directly use parameters from Ligandbook. For instance, in the future the Gromacs (Abraham *et al.*, 2015) topology generation tool `pdb2gmx` could query Ligandbook for parameters of small molecules that are not part of the included standard force field files and automatically create the simulation-ready topology for a protein with its ligand.

2 LICENSES

To credit the original parameters developers, upon creating a package users must accept to license the parameters under the CC BY-SA (Creative Commons Attribution-ShareAlike, `https://creativecommons.org/licenses/by-sa/2.0/`), which ensures that the parameters will always be available as Open Data (`https://okfn.org/opendata/`). Meta data is published under the CC0 1.0 Universal (CC0) Public Domain Dedication (`https://creativecommons.org/publicdomain/zero/1.0/`) and effectively puts these data into the public domain, which would be necessary for future enhancements to Ligandbook such as assigning DOIs for individual parameter sets.

3 SEARCHING

One of the most important functionalities in Ligandbook is the search. Standard text search can be used for broad queries that potentially yield hundreds of results. Text search with fields and boolean operators can narrow down results substantially. However, the chemical exact and substructure search sets Ligandbook apart from all other sites that provide parameter sets. It allows the user to precisely search by the chemistry of the compounds, as encoded by a chemical 2D structure. Search results are presented as a list of packages with the package name, a zoomable image of the compound structure and links to corresponding entries in the Protein Data Bank (PDB) (Berman *et al.*, 2000), PubChem (Bolton *et al.*, 2008) and Common Chemistry (`http://www.commonchemistry.org/`).

3.1 Text search

Simple text search All meta data fields are indexed and can be searched by entering search terms into the search field that is present on each page and also on the **Search** page. For instance, we can search for isobutylphenylpropanoic acid, a nonsteroidal anti-inflammatory drug better known as ibuprofen.

1. Enter *ibuprofen* in the search box and select *Search*.
2. A list with results is shown (Fig. 3). We find two hits: the (*S*) and (*R*) enantiomers of isobutylphenylpropanoic acid. The (*S*)-(+) isoform is the pharmacologically active compound and parameters are available in Ligandbook under packageId 1618.
3. Select the compound and package of interest and click on the *Show details* link to open the package view.
4. The package contains depictions at two levels of detail, meta data and links to other databases, and all versions of parameter files (Fig. 4).
5. Parameter files are downloaded by clicking the *Download* button.

Field search As an example for search by field, we show how a specific package, which is identified by the unique *packageId*, can be selected: Enter the field search

```
package.packageId:928
```

in the search box and the sole result is package 928 (a parametrization of benzene in the OPLS-AA force field).

For the RESTful API (see Section 1.4), the corresponding GET URL is

```
https://ligandbook.org/search/package.packageId:928/
    results.yml
```

(returning a YAML file).

3.2 Searching by chemical structure

A key feature of Ligandbook is the capability to search by chemical structure. This enables a user to accurately describe the chemistry of compounds instead of relying on text matches, which are rarely sufficiently precise. Let's assume we suspect that the active core of ibuprofen is represented by ethylphenylpropanoic acid. We can search for ibuprofen and related compounds by a *chemical substructure search* with the active core.

1. Under the **Search** menu, sketch the 2D structure in the Sketcher window (or enter the SMILES `C1=CC=CC=C1C(C(=O)O)C`), as shown in Fig. 5a. The chemical structures of common compounds can also be fetched from PubChem (Bolton *et al.*, 2008) by entering the compound name as a quoted string (e.g., `"ibuprofen"`).
2. Browse the list of results that are shown under the Sketcher window (Fig. 5b). As for the text search, we find the two enantiomers. Select the compound and parameter set of interest.

The most accurate way to search Ligandbook is to provide the query chemical structure in the Sketcher and to perform a search for an exact match. The Sketcher recognizes many compounds by name when entered into the SMILES text entry field as a quoted string. For example, ibuprofen can be entered as `"ibuprofen"`

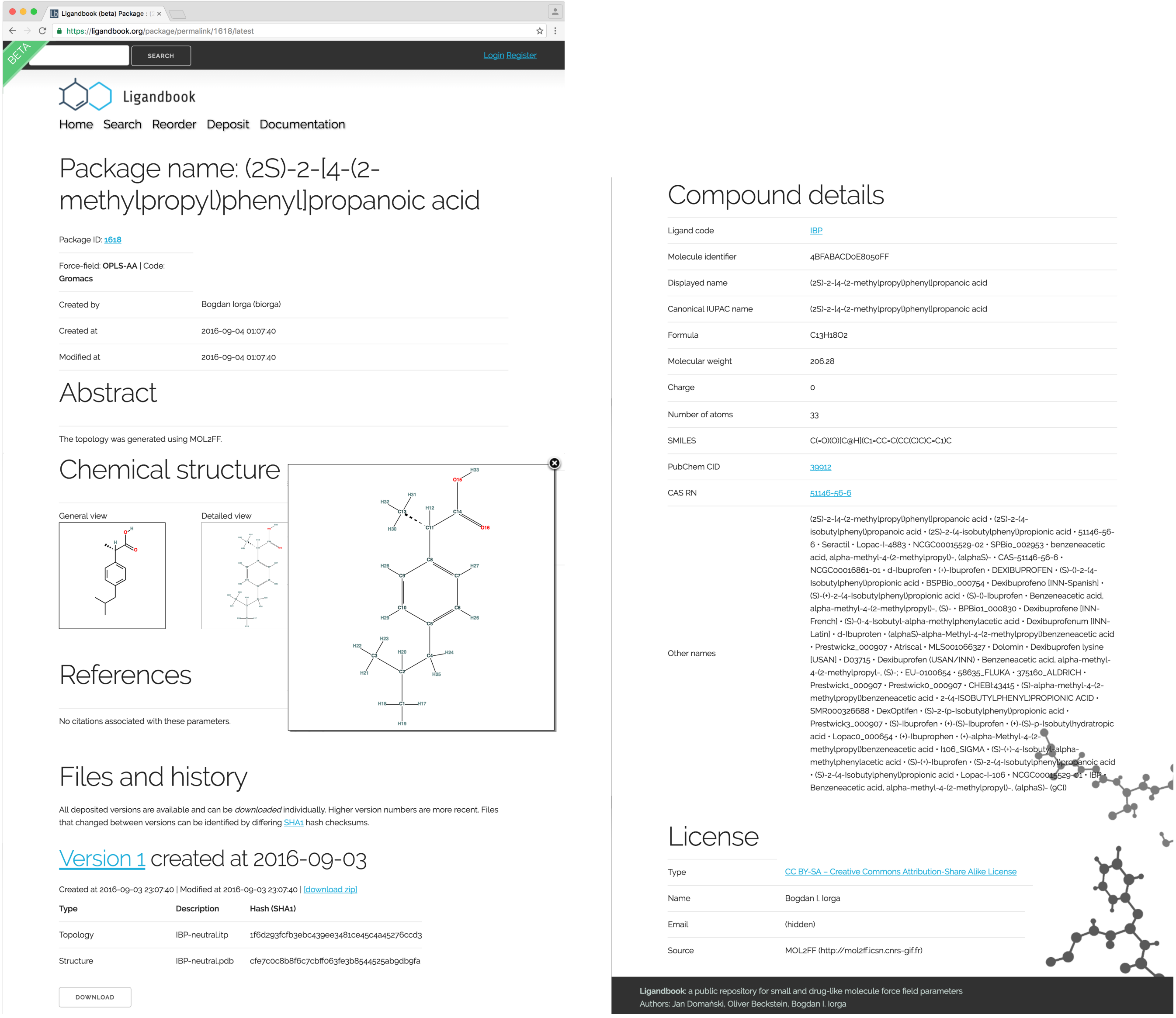

(a) Chemical structure and package details. (b) Compound details and License.

Fig. 4: Package view for package 1618 (ibuprofen). The detailed chemical structure is shown as a zoomed pop-out. Parameter files for version 1 can be downloaded. Meta data contains links (in blue) to other databases such as the Protein Data Bank with PDB Ligand code (IBP), PubChem with PubChem CID (39912) or the Common Chemistry database with the CAS RN (51146-56-6). The license type links to an explanation of what rights the license grants to the user.

and the Sketcher will immediately display the chemical structure. In conjunction with the *Exact search*, Ligandbook can be chemically accurately queried.

4 SETTING UP A MOLECULAR DYNAMICS SIMULATION

Ligandbook makes it easy to set up molecular dynamics simulations to probe protein-ligand interactions. As an example we show how to study the interaction of the anti-inflammatory drug aspirin with a potential target enzyme, phospholipase A2 (PLA2) (Burke and Dennis, 2009), using the Gromacs software package (Páll *et al.*, 2015; Abraham *et al.*, 2015). A crystal structure of aspirin in

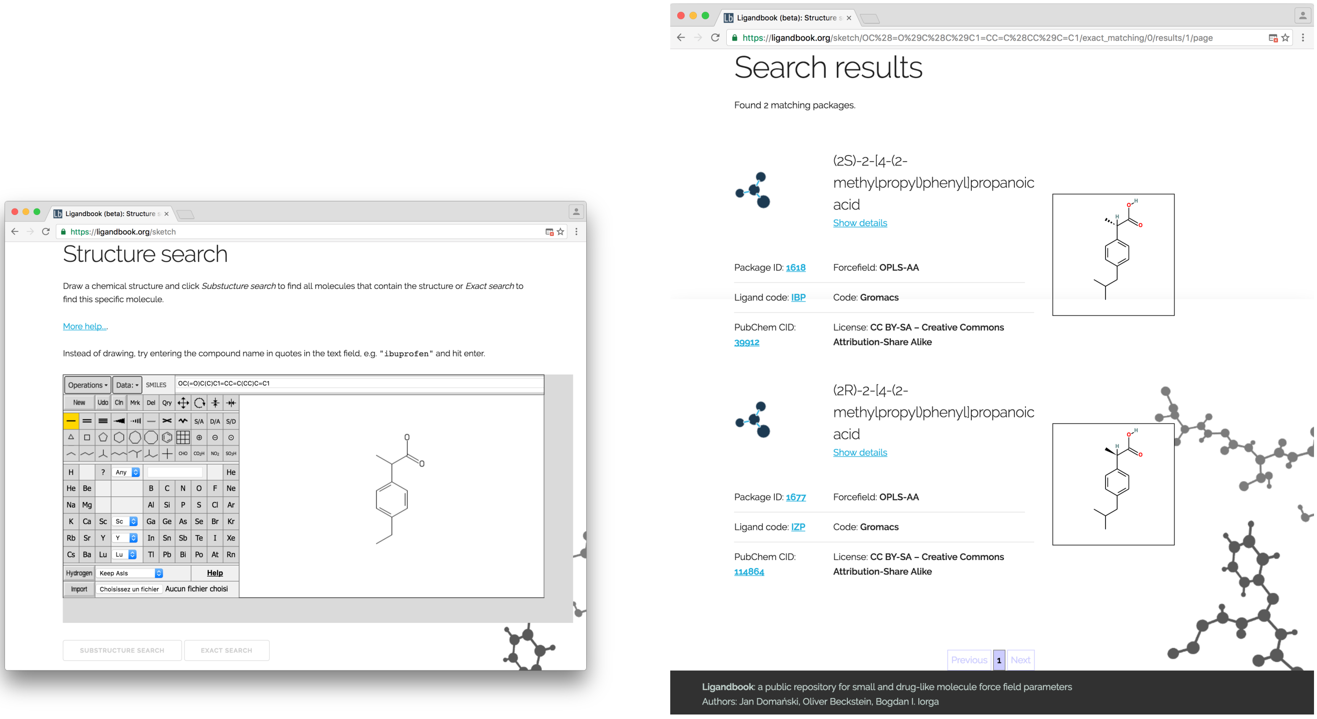

(a) Ethylphenylpropionic acid in the Sketcher window.

(b) Results for the chemical substructure search.

Fig. 5: Chemical substructure search for the pharmacological core of the drug ibuprofen. The 2D chemical structure can be drawn in the CACTVS Sketcher window or entered as a SMILES string. The deposited parameter sets can be searched either for an exact match (*Exact search*) or a substructure match (*Substructure search*), using the corresponding search buttons. Results are shown as a list of packages underneath the search window. Note that the GET URL string can also be used for programmatic access through the RESTful API (Section 1.4).

complex with PLA2 (PDB ID 1OXR) was solved to 1.9 Å resolution (Singh *et al.*, 2005).

Ligandbook contains two parameter sets for aspirin in the OPLS-AA force-field under packageIDs 785 (neutral form) and 2926 (anionic form). However the atom order of the parameter files is different from what is available in the PDB structures of the aspirin-protein complex. The user has to re-order the atoms in the structure to match the order in the topology file. This technical requirement is error-prone and tedious and creates an additional barrier to using parameters from a repository. Ligandbook overcomes this problem with its **Reorder** function that can re-order PDB structure files to match parameter files.

Ligand preparation We want to simulate the aspirin-PLA2 complex based on the 1OXR PDB file, which we can download directly from the PDB with the `wget` command (executed from the commandline):

```
wget http://files.rcsb.org/view/1OXR.pdb
```

Select out the ligand, resname AIN, using either a text editor, VMD (Humphrey *et al.*, 1996) or — as we do in this example — MDAnalysis (Michaud-Agrawal *et al.*, 2011; Gowers *et al.*, 2016) (in the Python interpreter):

```
from MDAnalysis import Universe
u = Universe("1OXR.pdb")
u.select_atoms("resname AIN").write("1oxr_resname-AIN.pdb")
u.select_atoms("protein or resname CA").write("1oxr_protein.pdb")
u.select_atoms("resname HOH").write("1oxr_xtalwater.pdb")
```

This writes out a ligand-only file `1oxr_resname-AIN.pdb` without any hydrogens (because PDB structures typically do not contain hydrogens); for later we also write out the protein (together with the calcium ion) and any crystal waters (`1oxr_protein.pdb` and `1oxr_xtalwater.pdb`).

In order to completely define the structure, the user should add hydrogens to the ligand and so completely determine the protonation state. For example, using Schrödinger's Maestro PrepWizard one could protonate the AIN ligand to obtain the anionic acetylsalicylate base (charge -1) and save it as the file `1oxr_resname-AIN_Hs.pdb`. (Alternatively, one could also use the open source RDKit (`http://www.rdkit.org/`) to protonate the ligand.) It is also possible to skip the protonation step and provide a structure without hydrogens. In this case, Ligandbook will add hydrogens and return all possible matches for different

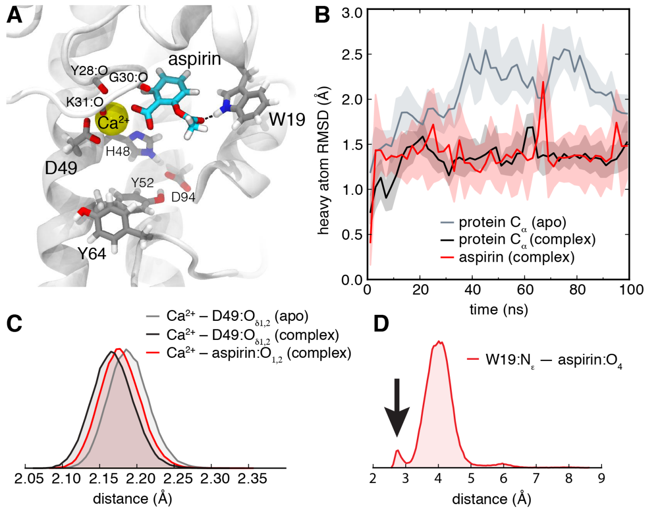

Fig. 6: Ligandbook enables simulations of protein-drug interactions. Example of a simulation with Ligandbook parameters for aspirin (2-acetyloxybenzoate, LBID 2926). **A** Phospholipase A2 (PLA2) with aspirin bound via calcium ion (yellow) and a hydrogen bond to W19. **B** Root mean square distance (RMSD) timeseries showing the apo enzyme (gray) and the PLA2-aspirin complex (black) as well as the RMSD for aspirin in the reference frame of the RMS-fitted protein (red). The complex shows smaller fluctuations than the free protein. **C** Probability distribution of the minimum distances between oxygen atoms and the calcium ion. **D** Probability distribution of the length of the hydrogen bond to W19. The arrow highlights the population of strong hydrogen bonds that is established towards the last ~10 ns of the 100 ns MD simulation.

forms of the query molecule but this approach can be error prone and for best results it is recommended to always manually protonate the query structure.

Under the **Reorder** menu, upload the ligand-only PDB (`1oxr_resname-AIN.pdb` or `1oxr_resname-AIN_Hs.pdb`) to get the re-orderd parameter files.

If the ligand was not protonated manually, Ligandbook will generate re-ordered parameters for all protonation states of the molecule in the database and show all matching packages. For a protonated ligand, only exactly matching structures will be shown. The user downloads the desired re-ordered and protonated structure (PDB format, e.g., `57b9d65c162e4.pdb`; note that file names are randomly generated for each uploaded compound) and corresponding topology file (Gromacs ITP format, e.g., `57b9d65c14721.itp`).

The ligand structure needs to be combined with a properly protonated protein structure (e.g., produced with the Gromacs 4.6.1 `pdb2gmx` tool (Páll *et al.*, 2015)):

```
pdb2gmx -f 1oxr_xtalwater.pdb -o 1oxr_xtalwater_H.pdb - ↩
     ignh -ff oplsaa -water tip4 -p water.top
pdb2gmx -f 1oxr_protein.pdb -o 1oxr_protein_H.pdb -ignh ↩
     -ff oplsaa -water tip4 -p system.top
```

(We also keep the crystal water molecules but because only oxygen atoms are stored for crystal waters, we must also add hydrogens.) Then merge the three components (here using MDAnalysis)

```
from MDAnalysis import Universe, Merge
protein = Universe("1oxr_protein_H.pdb").atoms
ligand = Universe("57b9d65c162e4.pdb").atoms
water = Universe("1oxr_xtalwater_H.pdb").atoms
u = Merge(protein, ligand, water)
u.atoms.write("1oxr_protein_AIN_xtalwater.pdb")
```

Note that the position in space of the re-ordered ligand structure is the same as the input and hence it is possible to simply combine protein and ligand. The fully protonated system is `1oxr_protein_AIN_xtalwater.pdb`; manually edit the `system.top` file by including the ligand ITP file with the line `#include "57b9d65c14721.itp"` and adding the ligand and the number of water molecules to the `[ molecules ]` section.

Further processing (solvating with water and ions, energy minimization, equilibration with position restraints) follows the standard Gromacs protocol, with the details summarized in the next section.

MD simulations Apo and drug-bound simulations of PLA2 were performed with Gromacs 4.6.1 (Páll *et al.*, 2015) using the OPLS-AA force field for proteins and ions (Kaminski *et al.*, 2001; Jensen and Jorgensen, 2006), and the TIP4P water model (Jorgensen *et al.*, 1983). The classical equations of motions were integrated with the leap-frog integrator with a time step of 2 fs. All bonds involving hydrogen atoms were constrained with P-LINCS (Hess, 2008) (order 4, 2 iterations). The neighbour list was updated every 5 steps. Simulations were performed at constant temperature $T = 300$ K using the Bussi velocity rescaling thermostat (Bussi *et al.*, 2007) (coupling time constant 0.1 ps) and constant pressure $P = 1$ bar with the isotropic Parrinello-Rahman barostat (Parrinello and Rahman, 1981) (coupling time constant 1 ps, compressibility 4.5×10^{-5} bar^{-1}). Long range electrostatic forces were computed with the SPME method (Essman *et al.*, 1995) (real space cutoff about 1 nm which was optimized by Gromacs on the fly to 1.502 nm, FFT grid spacing (started with 0.12 nm but was optimized to about 0.19 nm), 4th order spline) and Lennard-Jones interactions were cut off at 1 nm.

The protein-aspirin complex (PDB id 1OXR) was downloaded from the Protein Data Bank as described. Missing protein sidechains were added using the WHAT IF server (Vriend, 1990). The Ca^{2+} ion and all crystal water molecules were retained during system preparation. For the apo simulation, the bound aspirin molecule was removed; for the protein-drug complex simulation it was retained but the atoms were reordered to match the topology file from Ligandbook. The protein was solvated in a dodecahedral simulation cell with a minimum protein-box edge distance of 1.5 nm. The simulation systems contained NaCl for a free ion concentration of about 150 mM, comprising about 35,000 atoms in total.

Systems were energy minimized by alternating conjugate gradient and steepest descent algorithms until the maximum force in the system dropped below 1000 kJ $\cdot$ mol^{-1} $\cdot$ nm^{-1}. Protein (and drug) were position restrained (harmonic force constant 1000 kJ $\cdot$ mol^{-1} $\cdot$ nm^{-2}) for 1 ns so that the solvent could relax without disrupting the protein structure or protein-drug interactions seen in the crystal structures. Unrestrained equilibrium simulations started from the

final frame of the restrained simulation and were performed for 100 ns each.

Results The interaction of phospholipase A2 (PLA2) with aspirin has been described by Singh *et al.* (2005) on the basis of a 1.9-Å resolution crystal structure as a model of for interactions of proteins with non-steroidal anti-inflammatory drugs. We performed molecular dynamics (MD) simulations to better understand the difference between drug-protein interactions in a crystal structure and under physiological conditions.

Results are shown in Fig. 6. PLA2 in the apo state opens up somewhat compared to the crystal structure as indicated by the higher apo RMSD in Fig. 6B. The bound Ca^{2+} ion is only held in place by a tight interaction with the carboxylate group of D49 (Fig. 6C). In the drug-protein complex simulation, aspirin remained tightly bound to PLA2 over 100-ns simulation as shown by the low RMSD of the drug (Fig. 6B). Primarily, aspirin is held in place by the electrostatic interaction of its carboxylate group with the Ca^{2+} ion (Fig. 6C) that itself is tightly bound to PLA2 via D49 and the backbone carbonyl oxygens of Y28 (average distance 3.1 ± 0.9 Å) and K31 (3.8 ± 2.0 Å); G30:O, which contributes to the binding site in the crystal structure, flips out in the simulations (Fig. 6A). Aspirin effectively blocks access to the catalytic residues of PLA2, H48, Y52, and D94 (Burke and Dennis, 2009).

From the crystal structure an additional interaction with Y64 was described (distance 3.3 Å in 1OXR) (Singh *et al.*, 2005) but our simulations show this residue to be very mobile and the interaction not to be present under the conditions simulated here. However, a previously undetected interaction is established towards the end of the simulations by a hydrogen bond between the acetyl oxygen of aspirin and the nitrogen of W19 (Fig. 6A, D). The initial W19:N_ϵ–aspirin:O_4 distance is about 4 Å, corresponding to a weak hydrogen bond. However, towards the end of the simulation, the distance shrinks to < 3 Å, which is indicative of the formation of a strong hydrogen bond.

These preliminary simulations indicate that the interactions between aspirin and PLA2 are qualitatively different in the crystal conditions and the physiological conditions present in the MD simulations. Future work would need to further validate the parameters for aspirin and investigate the robustness of the observed interactions under repeated and longer simulations.

This example shows how Ligandbook enables the study of drug-protein interactions by removing a substantial barrier to setting up the simulation system.

REFERENCES

Abraham, M. J., Murtola, T., Schulz, R., Páll, S., Smith, J. C., Hess, B., and Lindahl, E. (2015). GROMACS: High performance molecular simulations through multi-level parallelism from laptops to supercomputers. *SoftwareX*, **1–2**, 19–25.

Berman, H., Westbrook, J., Feng, Z., Gilliland, G., Bhat, T., Weissig, H., Shindyalov, I., and Bourne, P. (2000). The Protein Data Bank. *Nucleic Acids Res.*, **28**(1), 235–242 (http://www.pdb.org/).

Bolton, E., Wang, Y., Thiessen, P., and Bryant, S. (2008). PubChem: Integrated platform of small molecules and biological activities. *Ann. Rep. Comput. Chem.*, **4**, 217–241 (http://pubchem.ncbi.nlm.nih.gov/).

Burke, J. E. and Dennis, E. A. (2009). Phospholipase A2 structure/function, mechanism, and signaling. *J Lipid Res*, **50 Suppl**, S237–42.

Bussi, G., Donadio, D., and Parrinello, M. (2007). Canonical sampling through velocity rescaling. *J. Chem. Phys.*, **126**(1), 01410.

Essman, U., Perela, L., Berkowitz, M. L., Darden, T., Lee, H., and Pedersen, L. G. (1995). A smooth particle mesh Ewald method. *J. Chem. Phys.*, **103**, 8577–8592.

Gowers, R. J., Linke, M., Barnoud, J., Reddy, T. J. E., Melo, M. N., Seyler, S. L., Dotson, D. L., Domański, J., Buchoux, S., Kenney, I. M., and Beckstein, O. (2016). MDAnalysis: A Python package for the rapid analysis of molecular dynamics simulations. In *Proceedings of the 15th Scientific Computing with Python Conference (SciPy 2016)*, Austin, TX.

Hess, B. (2008). P-LINCS: A parallel linear constraint solver for molecular simulation. *J. Chem. Theory Comput.*, **4**(1), 116–122.

Humphrey, W., Dalke, A., and Schulten, K. (1996). VMD – Visual Molecular Dynamics. *J. Mol. Graph.*, **14**, 33–38.

Ihlenfeldt, W., Takahashi, Y., Abe, H., and Sasaki, S. (1994). Computation and management of chemical properties in CACTVS: An extensible networked approach toward modularity and compatibility. *J. Chem. Inf. Comput. Sci.*, **34**(1), 109–116 (http://www.xemistry.com/).

Ihlenfeldt, W., Bolton, E., and Bryant, S. (2009). The PubChem chemical structure sketcher. *J. Cheminform.*, **1**, 20.

Jensen, K. P. and Jorgensen, W. L. (2006). Halide, ammonium, and alkali metal ion parameters for modeling aqueous solutions. *J. Chem. Theory Comput.*, **2**(6), 1499–1509.

Jorgensen, W. L., Chandrasekhar, J., Madura, J. D., Impey, R. W., and Klein, M. L. (1983). Comparison of simple potential functions for simulating liquid water. *J. Chem. Phys.*, **79**(2), 926–935.

Kaminski, G. A., Friesner, R. A., Tirado-Rives, J., and Jorgensen, W. L. (2001). Evaluation and reparametrization of the OPLS-AA force field for proteins via comparison with accurate quantum chemical calculations on peptides. *J. Phys. Chem. B*, **105**(28), 6474–6487.

McCandless, M., Hatcher, E., and Gospodnetic, O. (2010). *Lucene in Action, Second Edition: Covers Apache Lucene 3.0*. Manning Publications.

Michaud-Agrawal, N., Denning, E. J., Woolf, T. B., and Beckstein, O. (2011). MDAnalysis: A toolkit for the analysis of molecular dynamics simulations. *J. Comp. Chem.*, **32**, 2319–2327.

Páll, S., Abraham, M. J., Kutzner, C., Hess, B., and Lindahl, E. (2015). Tackling exascale software challenges in molecular dynamics simulations with GROMACS. In S. Markidis and E. Laure, editors, *Solving Software Challenges for Exascale: International Conference on Exascale Applications and Software, EASC 2014, Stockholm, Sweden, April 2-3, 2014, Revised Selected Papers*, volume 8759 of *Lecture Notes in Computer Science*, pages 3–27. Springer International Publishing, Switzerland.

Parrinello, M. and Rahman, A. (1981). Polymorphic transitions in single crystals: A new molecular dynamics method. *J. Appl. Phys.*, **52**(12), 7182–7190.

Singh, R. K., Ethayathulla, A. S., Jabeen, T., Sharma, S., Kaur, P., and Singh, T. P. (2005). Aspirin induces its anti-inflammatory effects through its specific binding to phospholipase A2: crystal structure of the complex formed between phospholipase A2 and aspirin at 1.9 Å resolution. *J. Drug Target.*, **13**(2), 113–119.

Vriend, G. (1990). WHAT IF: A molecular modeling and drug design program. *J. Mol. Graph.*, **8**, 52–56.
